# Supplementary material for: Antiproliferative Effect of Acridine Chalcone Is Mediated by Induction of Oxidative Stress
Source: Biomolecules. 2020 Feb 22;10(2):345. doi: 10.3390/biom10020345 (PMC7072140; doi:10.3390/biom10020345)
Supplement: Supplementary file 1 [file biomolecules-10-00345-s001.pdf]

biomolecules

MDPI

Supplementary Materials

Antiproliferative Effect of Acridine Chalcone is Mediated by Induction of Oxidative Stress

Peter Takac [1,2], Martin Kello [1,*], Maria Vilkova [3], Janka Vaskova [4], Radka Michalkova [1], Gabriela Mojzisova [5] and Jan Mojzis [1,*]

[1] Department of Pharmacology, Faculty of Medicine, Pavol Jozef Safarik University, 04011 Kosice, Slovak Republic
[2] Institute of Human and Clinical Pharmacology, University of Veterinary Medicine and Pharmacy, 041 81 Košice, Slovak Republic
[3] Department of Organic chemistry, Faculty of Science, Pavol Jozef Safarik University, 040 01, Kosice, Slovak Republic
[4] Department of Medical and Clinical Biochemistry, Faculty of Medicine, Pavol Jozef Safarik University, 040 01, Kosice, Slovak Republic
[5] Department of Experimental Medicine, Faculty of Medicine, Pavol Jozef Safarik University, 040 01, Kosice, Slovak Republic
* Correspondence: jan.mojzis@upjs.sk (J.M.); kellomartin@yahoo.com (M.K.)

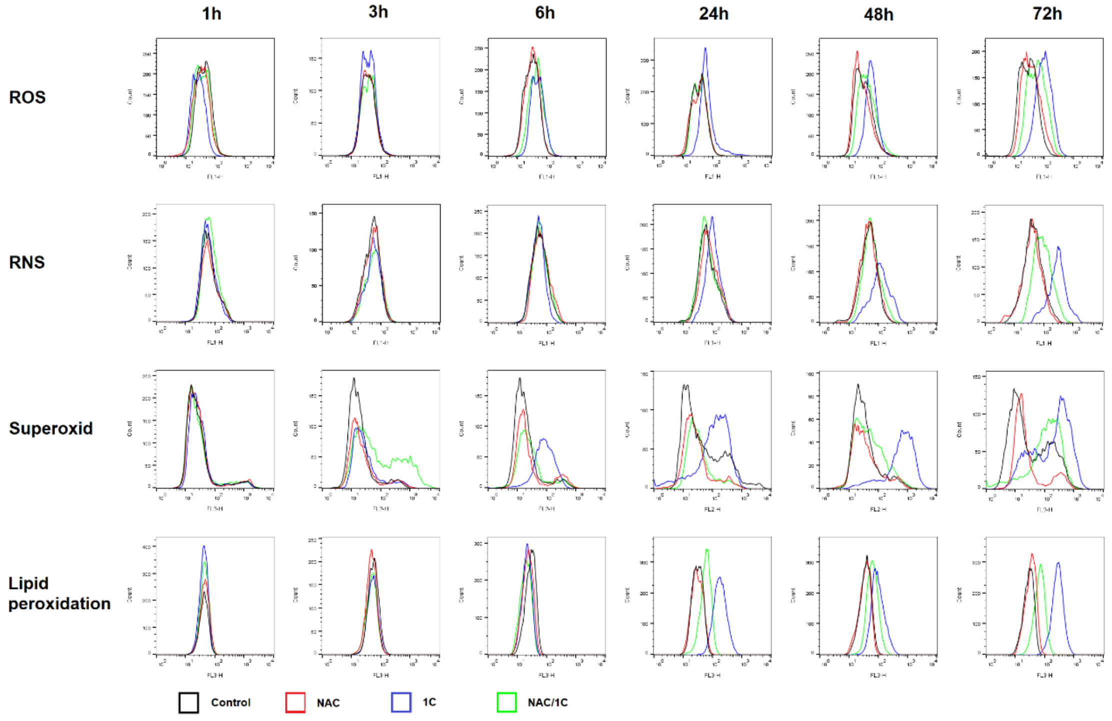

Figure S1. Representative flow cytometry histograms of free radicals production in HCT116 cells after 1C and NAC/1C treatment.

Table S1. Antioxidant status of HCT116 cells after 1C, NAC and NAC/1C treatment. The influence of 1C and combination NAC/1C on glutathione content, GPx activity, GR activity and GST activity—average from 3 experiments

Group	Time (h)	GPx (µkat/mg prot)	GR (kat/kg prot)	GST (µkat/mg prot)	GSH (nmol SH/mg prot)
Control	1	0.5746 ± 0 0821	16.30 ± 3.22	0.0856 ± 0.0242	0.6776 ± 0.3111
	3	0.4545 ± 0 1088	14.71 ± 2.83	0.1221 ± 0.0247	1.6866 ± 0.6344
	6	0.5228 ± 0.1435	5.59 ± 0.70	0.0953 ± 0.0368	0.6151 ± 0.3404
	24	0.1507 ± 0.0440	8.09 ± 2.45	0.0865 ± 0.0111	1.0253 ± 0.5946
	48	0.4392 ± 0.0404	4.75 ± 0.98	0.1267 ± 0.0672	0.6270 ± 0.1267
	72	0.1684 ± 0.0527	12.99 ± 0.75	0.3326 ± 0.0344	1.1079 ± 0.5781
NAC	1	0.2678 ± 0.0675	7.89 ± 1.95	0.4530 ± 0.0136	0.5056 ± 0.0701
	3	0.2275 ± 0.0214	8.92 ± 4.33	0.8030 ± 0.1711	1.8669 ± 0.4320
	6	0.1055 ± 0.0500	2.66 ± 1.71	0.6833 ± 0.3870	1.2839 ± 0.2253
	24	0.4081 ± 0.0854	18.45 ± 5.28	0.9982 ± 0.0533	0.1647 ± 0.5342
	48	0.3129 ± 0.0835	3.21 ± 1.13	0.7002 ± 0.0109	0.5777 ± 0.1679
	72	0.2397 ± 0.0120	4.38 ± 2.10	0.8892 ± 0.0712	1.2166 ± 0.2319
1C	1	0.6795 ± 0.0768	12.59 ± 2.83	1.2325 ± 0.0570	0.6711 ± 0.1992
	3	0.4904 ±.0.0605	9.47 ± 3.41	1.0385 ± 0.0658	1.2435 ± 0.2480
	6	0.5179 ± 0.1032	8.72 ± 3.47	1.5965 ± 0.4384	1.4827 ± 0.2931
	24	0.5057 ± 0.1070	6.68 ± 1.51	1.2543 ± 0.0308	0.3579 ± 0.0422
	48	0.3593 ± 0.0578	6.56 ± 1.19	1.3765 ± 0.0729	1.5355 ± 0.5354
	72	0.1952 ± 0.0764	7.88 ± 3.00	1.4337 ± 0.0596	1.0698 ± 0.0818
1C/NAC	1	0.4105 ± 0.1183	8.42 ± 1.52	1.0045 ± 0.2931	1.2218 ± 0.1276
	3	0.2403 ± 0.0252	12.75 ± 1.95	0.7020 ± 0.0368	0.5259 ± 0.0378
	6	0.3459 ± 0.0653	5.78 ± 1.95	0.7257 ± 0.0108	1.5086 ± 0.0444
	24	0.4895 ± 0.0298	5.97 ± 2.59	0.8849 ± 0.1119	0.2516 ± 0.0209
	48	0.4685 ± 0.0841	10.91 ± 0.88	0.9109 ± 0.0745	1.2389 ± 0.1314
	72	0.3483 ± 0.0645	7.98 ± 1.13	1.1589 ± 0.0937	1.1988 ± 0.1204
